# The Binocular Balance at High Spatial Frequencies as Revealed by the Binocular Orientation Combination Task

**DOI:** 10.3389/fnhum.2019.00106

**Published:** 2019-04-02

**Authors:** Yonghua Wang, Zhifen He, Yunjie Liang, Yiya Chen, Ling Gong, Yu Mao, Xiaoxin Chen, Zhimo Yao, Daniel P. Spiegel, Jia Qu, Fan Lu, Jiawei Zhou, Robert F. Hess

**Affiliations:** ^1^The First Affiliated Hospital of Wenzhou Medical University, Wenzhou, China; ^2^School of Ophthalmology and Optometry and Eye hospital, Wenzhou Medical University, Wenzhou, China; ^3^State Key Laboratory of Ophthalmology, Optometry and Vision Science, Wenzhou Medical University, Wenzhou, China; ^4^Vision Sciences, Essilor R&D, Center for Innovation and Technology, Singapore, Singapore; ^5^McGill Vision Research, Department of Ophthalmology, McGill University, Montreal, QC, Canada

**Keywords:** binocular eye dominance, spatial frequency, binocular orientation combination, binocular phase combination, contrast-gain

## Abstract

How to precisely quantify the binocular eye balance (i.e., the contribution that each eye makes to the binocular percept) across a range of spatial frequencies using a binocular combination task, is an important issue in both clinical and basic research. In this study, we aimed to compare the precision of a binocular orientation combination paradigm with that of the standard binocular phase combination paradigm in measuring the binocular eye balance at low to high spatial frequencies. Nine normal adults (average age: 24.6 ± 2.0 years old) participated. Subjects viewed an LED screen dichoptically with polarized glasses in a dark room. The method of constant stimuli was used to quantitatively assess the point of subjective equality (PSE), i.e., the interocular contrast ratio when two eyes are balanced in binocular combination, for stimulus spatial frequencies from 0.5 to 8 cycles/degree. Precision was quantified by the variance [i.e., standard error (SE), obtained from 100 bootstrap estimates] associated to the PSE. Using stimuli whose interocular phase difference at the edge of the gratings was matched at 45°, we found that the orientation paradigm provides more precision than the standard binocular phase combination paradigm, especially at high frequencies (Experiment 1). Such differences remained when using stimuli that had three times larger interocular phase difference (Experiment 2) or displayed at four times higher stimuli resolution (Experiment 3). Our results indicate that a binocular combination tasked based on orientation rather than phase, provides a more precise estimate of binocular eye balance in human adults at high spatial frequencies, thus allowing a binocular balance to be assessed within the spatial region where amblyopes are most defective (i.e., high spatial frequencies).

## Introduction

Binocularity is an important visual property in primates. In humans, there are a number of conditions that involve disruptions to normal binocular vision and these are associated with gross changes in eye balance. For example, strabismus (Feng et al., 2015), anisometropia (Zhou et al., 2016) and amblyopia (Huang et al., 2009, 2011; Ding et al., 2013a; Zhou et al., 2013; Kwon et al., 2014) are all associated with large interocular imbalances, in which one eye contributes much more than the other to binocular processing. To simplify, we quantified the contribution that each eye makes to the binocular percept in terms of the “binocular eye balance” in this article.

Several groups have worked on developing binocular therapies to re-balance the two eyes, for example, the anti-suppression training (Li et al., 2013), the push-pull training (Ooi et al., 2013), the binocular action video game training (Gambacorta et al., 2018), the altered-reality based dichoptic training (Bao et al., 2018), for review, see Hess and Thompson (2015). Therefore, to precisely measure the binocular eye balance is important not only for understanding the binocular visual deficits in these above-mentioned visual diseases but also for designing and assessing binocular treatments to improve patients’ binocular function.

The binocular eye balance has been quantitatively assessed either when the two eyes view very different monocular patterns that produce perceptual rivalry (Ooi and He, 2001; Handa et al., 2004, 2006, 2012; Kwon et al., 2015) or when the two eyes view similar monocular patterns that fuse into a single cyclopean percept (Ding and Sperling, 2006; Huang et al., 2010; Zhou et al., 2014a). The latter approach is of particular interest to us as it reflects the normal state of affairs when the two eyes work together in binocular perception. Over the last decade, several paradigms have been introduced to study the nature of binocular combination, including the use of stimulus phase (Ding and Sperling, 2006; Huang et al., 2009), contrast (Huang et al., 2010; Ding et al., 2013b), second-order modulation (Zhou et al., 2014a,b), global motion coherence (Hess et al., 2007) and global orientation coherence (Zhou et al., 2013). The basic principle behind these methods is that the interocular contrast ratio is varied until the two eyes contribute equally to binocular vision, the interocular contrast ratio so measured can be used to quantify the binocular eye balance.

Recently, (Spiegel et al., 2016; Yehezkel et al., 2016) presented data to show that human adults are able to fuse two slightly differently orientated monocular gratings when the interocular orientation difference is less than 20°. Yehezkel et al. (2016) also demonstrated that the binocular combination of stimulus orientation is also contrast-gain controlled. The dependence on contrast-gain control suggests that it may provide an important new approach for quantifying perceptual eye dominance. Only a medium spatial frequency (i.e., 3 cycles/degree) was tested by Yehezkel et al. (2016), so we wanted to validate this approach for higher spatial frequencies as our understanding of perceptual eye balance in normals and the suppressive influences that disturb this balance in amblyopes and other binocular disorders is lacking in the high spatial frequency range.

**Figure 1:**
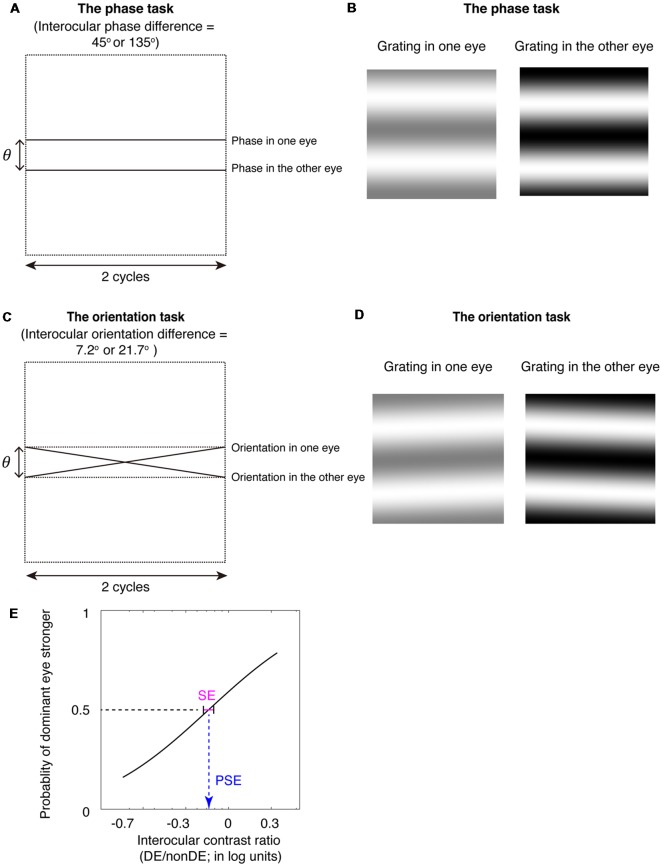
The binocular phase and orientation combination tasks. **(A)** Diagrammatic illustration of the binocular phase combination task. In Experiments 1 and 3, the interocular phase-shift difference was set as 45°; In Experiment 2, the interocular phase-shift difference was set as 135°. **(B)** The two horizontal gratings that we dichoptically presented to the two eyes in the binocular phase combination task. The contrast of the gratings in the nondominant eye was fixed at 50%, while the contrast of the gratings in the dominant eye was varied with an interocular contrast ratio (0.2, 0.5, 0.8, 1.1, 1.4, 1.7, and 2.0); subjects were instructed to answer whether the dark stripe of the perceived cyclopean grating was above or below the center of the screen by pressing a keyboard. **(C)** Diagrammatic illustration of the binocular orientation combination task. In Experiments 1 and 3, the interocular orientation difference was set as 7.2°; which enabled a 45° of interocular phase difference at the edge of the two-cycle gratings; In Experiment 2, the interocular orientation difference was set as 21.7°; which enabled a 135° of interocular phase difference at the edge of the two-cycle gratings. **(D)** The two oriented gratings that we dichoptically presented to the two eyes in the binocular orientation combination task. The contrast of the gratings in the nondominant eye was fixed at 50%, while the contrast of the gratings in the dominant eye was varied with an interocular contrast ratio (0.2, 0.5, 0.8, 1.1, 1.4, 1.7, and 2.0); subjects were instructed to answer whether the perceived cyclopean grating was rotated clockwise or counter-clockwise relative to the horizontal by pressing a keyboard. **(E)** An illustration of the psychometric function. The proportion of trials in which the observers reported that dominant eye dominated (i.e., the perceived orientation or the perceived phase closed to the input orientation or phase in the dominant eye) was plotted as a function of the interocular contrast ratio (DE/nonDE; in log units). We fitted this curve using the cumulative Gaussian distribution function. The “point of subjective equality (PSE)” (i.e., the PSE) corresponds to the 50% point of the best fitting Gaussian function was derived from the fitting, which indicates the point at which the two eyes were balanced in binocular combination. The standard error (SE) of the estimated “PSE” were derived based on 100 times’ parametric bootstrapping procedure and used as the precision of the measure.

To be able to extend measurements of binocular eye balance to higher spatial frequencies while using interocular contrast with its contrast-gain control underpinnings, unlike Yehezkel et al. (2016), we used gratings not rotated about the vertical meridian but rotated about the horizontal meridian (Figures 1C,D; Spiegel et al., 2016). This enables us to provide the appropriate comparison with a standard binocular phase combination task (Ding and Sperling, 2006) that utilizes horizontal stimuli (Figures 1A,B) and to avoid inducing a depth percept as a consequence of the interocular phase or orientation differences. The binocular phase combination was developed by Ding and Sperling (2006), in which two horizontally oriented sine-wave gratings with phase-shifts in opposite directions of the same magnitude were presented dichoptically, the interocular imbalance being quantified by the interocular contrast ratio corresponding to a binocular perceived phase of 0° (i.e., when the two eyes had balanced contributions). It has been widely used, by us and other groups, in quantifying the binocular eye balance in normals and patients with binocular disorders (Ding and Sperling, 2006; Huang et al., 2009, 2010, 2011; Ding et al., 2013a,b; Zhou et al., 2013, 2014a,b, 2016; Kwon et al., 2014; Feng et al., 2015).

Furthermore, we used a method of constant stimuli to measure the binocular eye balance for a binocular combination of orientation information, rather than using a signal detection rating method, as Yehezkel et al. (2016) did. The binocular eye balance was quantified by the 50% point of the best fitting psychometric function, i.e., the point of subjectively equality (PSE). The use of the method of constant stimuli enables one to derive non-parametric bootstrapped estimates of the precision of binocular eye balance measurements [i.e., using the psychometric data; standard error (SE) on the PSE] for different the spatial frequencies that can be compared with that of the standard binocular phase combination task (Figure 1E). The advantage of comparing results from the binocular orientation paradigm with that from the binocular phase combination paradigm is that both methods use comparable stimuli and are based on a common contrast-gain control model for binocular combination (Ding and Sperling, 2006; Yehezkel et al., 2016). Previous studies have shown that the probability of fusion decreased as the interocular phase difference increased (Georgeson and Wallis, 2014; Spiegel et al., 2016), we thus used matched spatial phase-shifts at the edges of the stimuli (Figures 1A,C) to rule out any potential binocular rivalry artifact in the comparison of these two tasks. We found that the binocular orientation combination paradigm provided more precise estimates (smaller SE of the PSE) than that of the binocular phase combination paradigm, especially at high spatial frequencies. Our results suggest that the binocular orientation combination paradigm is a more precise tool than that of the binocular phase combination paradigm to quantify binocular eye balance at high spatial frequencies.

## Materials and Methods

### Participants

Nine adults (average age: 24.6 ± 2.0 years old) with normal or corrected to normal visual acuity (LogMAR ≤ 0.0) in the two eyes and normal stereopsis (less than 60 arc seconds in the Stereo Fly Test; Stereo Optical Company, Inc., Chicago, IL, USA) participated in this study. All subjects had normal alignment and no history of ocular operations or any organic eye diseases. Observers wore their habitual optical correction if required.

Except for the first author (YW), all observers were naive as to the purpose of the experiment. Informed consent was obtained prior to the study, which was approved by the Institutional Review Board of the Wenzhou Medical University in China. The methods were carried out in accordance with the approved guidelines.

### Apparatus

All measurements were conducted on a PC computer running Matlab (MathWorks, Inc., Natick, MA, USA) with Psychtoolbox 3.0.9 extensions (Brainard, 1997; Pelli, 1997). The stimuli were presented on a gamma-corrected LG D2341PY 3D LED screen (LG Life Science, South Korea) with a 1,920 × 1,080 resolution and a 60 Hz refresh rate. Subjects viewed the display dichoptically with polarized glasses in a dark room at a viewing distance of 85.5 cm in Experiments 1 and 2 and 342 cm in Experiment 3. The change in viewing distance was used to control for any loss in sensitivity in the phase combination task due to loss of screen resolution (i.e., pixels/spatial cycle). The background luminance was 39.4 cd/m^2^ on the screen and 16.25 cd/m^2^ through the polarized glasses. The polarized glasses had low crosstalk between the two lenses (<3% at the highest contrast level).

### Design

In Experiment 1, observers’ binocular eye balance probability in binocular orientation combination as well as binocular phase combination was measured at seven interocular contrast ratios (0.2, 0.5, 0.8, 1.1, 1.4, 1.7, and 2.0) for five spatial frequencies (0.5, 1, 2, 4 and 8 cycles/degree) using the method of constant stimuli. The interocular phase-shift difference was 45° at the edge of the gratings for both tasks. Different tasks (phase and orientation) and spatial frequencies (in total 10 sessions for 2 tasks × 5 spatial frequencies) were measured in different measurement sessions with a randomized order across subjects. In Experiment 2, similar measures as Experiment 1 were done at 8 cycles/degree with the interocular phase-shift difference at the edge of the gratings increased to 135°. In Experiment 3, similar measures as Experiment 1 were done at 8 cycles/degree with the viewing distance increased to 342 cm.

In one typical measurement session of the phase task or the orientation task, two configurations were used for each interocular contrast ratio to cancel any potential positional starting bias: for the phase task, the phase-shift was +22.5° from horizontal (in Experiments 1 and 3) or +65.5° from horizontal (in Experiment 2) in one eye and −22.5° from horizontal (in Experiments 1 and 3) or −65.5° from horizontal (in Experiment 2) in the other eye in one configuration and in the other, the reverse; For the orientation task, the orientation was +3.6° (in Experiments 1 and 3) or +10.85° (in Experiment 2) counter-clockwise towards horizontal position in one eye and −3.6° (in Experiments 1 and 3) or −10.85° (in Experiment 2) clockwise toward horizontal position in the other eye in one configuration and in the other, the reverse. Each condition (interocular contrast ratio and stimuli configuration) was repeated 40 times (i.e., 80 trials per interocular contrast ratio). In total, there were 560 trials in each measurement session for a given task and a given spatial frequency; the interocular contrast ratio and configuration were randomized in different trials. Each measurement session was divided into four sub-blocks (i.e., 140 trials) in the measurement, observers were allowed to take short breaks after they finished one sub-block if they felt tired.

Before the start of data collection, a hole-in-the-hand test (Dane and Dane, 2004) was used to determine sighting dominance; proper demonstrations were provided with practice trials to ensure observers understood the task.

### Stimuli

As illustrated in Figure 1, stimuli for the binocular phase combination task were two horizontally oriented sine-wave gratings, with equal and opposite phase-shifts relative to the horizontal center. Stimuli for the binocular orientation task were two horizontally oriented sine-wave gratings, with equal and opposite orientation tilts relative to the horizontal center. A fixed two cycles of gratings were used when testing different spatial frequencies. The contrast of the gratings in the nondominant eye was fixed at 50%, while the contrast of the gratings in the dominant eye was varied with an interocular contrast ratio (0.2, 0.5, 0.8, 1.1, 1.4, 1.7, and 2.0). A high-contrast frame (length: 3*gratings size) with four white diagonal bars (length: 1.4*gratings size) were presented continuously surrounding the grating in each eye to assist observers in maintaining vergence when they performed the tasks. The size of the grating and frame were varied proportionately at different spatial frequencies.

### Procedure

In each measurement session, subjects first completed an alignment task in which they adjusted the coordinates of stimuli with cross and dots to make sure the images seen by the two eyes were perfectly fused. This was followed by the binocular phase combination or the binocular orientation combination task. In one typical trial of the two tasks, the gratings were presented continually until subjects made their decision by pressing a corresponding keyboard and then followed by a 500-ms blank screen (with only the surrounding frame and diagonal bars presented) and the presentation of the next trial. For the binocular phase combination task, subjects were instructed to answer whether the dark stripe of the perceived cyclopean grating was above or below the center of the screen by pressing a keyboard. For the binocular orientation combination task, subjects needed to answer whether the perceived cyclopean grating was rotated clockwise or counter-clockwise relative to the horizontal by pressing a keyboard.

### Curve Fits

We fitted the proportion of trials in which the observers reported that dominant eye dominated (i.e., the perceived orientation or the perceived phase closed to the input orientation or phase in the dominant eye) vs. interocular contrast ratio (DE/nonDE) using cumulative Gaussian distribution functions. Two free parameters were derived from the fitting of these cumulative Gaussian distribution functions, namely the “*PSE*” and the “*Sigma*”: the “*PSE*” corresponds to the 50% point of the best fitting Gaussian function, i.e., the PSE, indicates the point at which the two eyes were balanced in binocular combination. The “*Sigma*” is a measure of the slope at the *PSE* point. The SEs of the estimated “*PSE*” were derived based on 100 times’ parametric bootstrapping procedure and used as the precision of the measure. For each psychometric function, what we did was: (1) fit the observed data using the cumulative Gaussian function; (2) resample to get 100 groups of data. In particular, for each interocular contrast ratio, we resampled 100 times based on the binomial distribution *binornd(n, p)*, in which *n* was the trail number we measured in this study for each interocular condition (*n* = 80) and *p* was estimated from the cumulative Gaussian function derived from the fitting of the observed data and (3) fit the 100 groups of data using the cumulative Gaussian functions. The standard deviation of the 100 fitted *PSEs* based on the resampled data was used as the SE of the estimated *PSE* based on the observed data.

## Results

### Experiment 1. Measurement of Binocular Eye Balance From Low To High Spatial Frequencies

**Figure 2:**
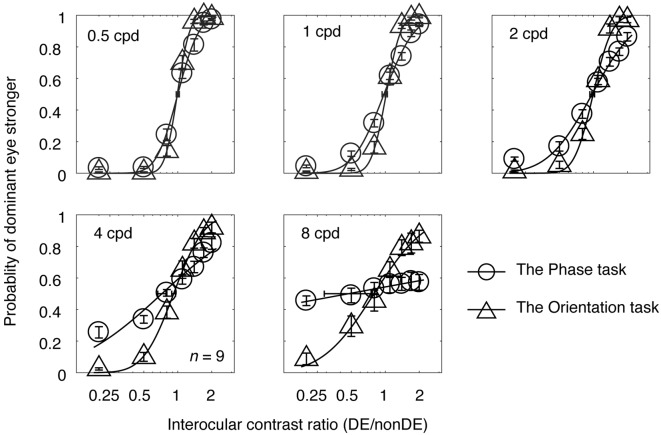
The average psychometric functions for the two tasks. Data of different spatial frequencies are shown in separate panels. In each panel, the mean probability of the dominant eye stronger was plotted as a function of interocular contrast ratio (DE/non-DE) for the two tasks. The solid and dash line represents the best fitting cumulative distribution functions for the two tasks. Error bars represent SEs across the nine subjects. The horizontal error bars in each graph indicate the derived PSE (the PSE, i.e., the point at which the two eyes were balanced in binocular combination) and its bootstrapped SEs (based on 100 times’ parametric bootstrapping procedure).

In Experiment 1, observers’ psychometric functions were measured with an interocular phase difference at the edge of the grating being set as 45° from 0.5 to 8 cycles/degree at a viewing distance of 85.5 cm. The averaged psychometric functions for the nine subjects are presented in Figure 2, in which each panel contains results for the two tasks at one spatial frequency. The derived PSE estimate (the PSE, i.e., the point at which the two eyes were balanced in binocular combination) with its bootstrapped SE (based on 100 times’ parametric bootstrapping procedure) were also provided for the two tasks in each panel. Apparently, as spatial frequency increases, the slopes of both curves gradually decreased, especially at higher spatial frequencies (4 and 8 cycles/degree). Also, the slope of the psychometric function for the orientation task was steeper than that for the phase task at all, but especially higher, spatial frequencies. This shows that the orientation task provides a more precisie estimate of the binocular eye balance than the phase task, especially at higher spatial frequencies.

**Figure 3:**
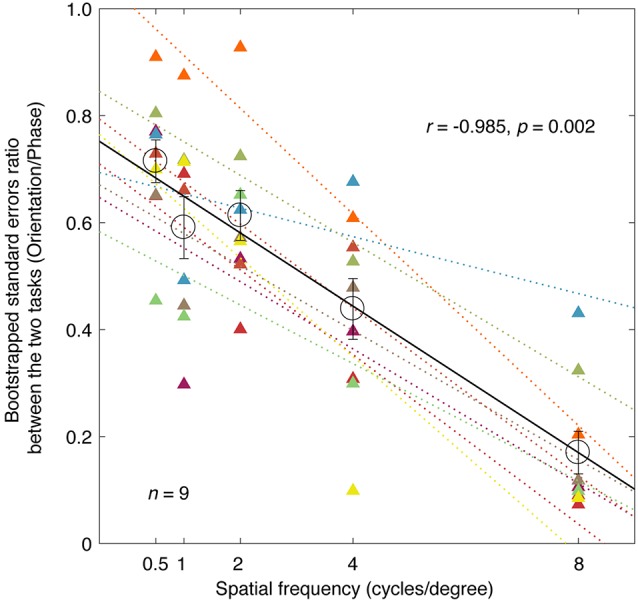
A comparison of the precision of the two tasks; the relationship between the ratio of the bootstrapped SEs and spatial frequency. Each circle represents an averaged SE ratio (orientation/phase) of the PSE across nine subjects at one spatial frequency. Individuals’ results are also provided for each spatial frequency using triangle symbols. The best linear fits for individuals and means are provided with colored dashed lines and black solid line, respectively. Regression analysis results are provided in the figure. Error bars represent SEs across the nine subjects.

To highlight the difference in precision between the two tasks, we plotted the averaged bootstrapped SE ratio between the two tasks (orientation/phase) as a function of spatial frequency in Figure 3 (individuals’ results are also provided). The orientation measurements exhibited a smaller SE than the corresponding phase measurements at all the spatial frequencies we tested here, as the sigma ratio was always less than 1 (all *p* < 0.001, two-tailed one sample *t*-test compared to 1). This result indicated that the orientation paradigm has higher precision for measuring the binocular eye balance. It is also obvious that as spatial frequency increases, the SE ratio decreased, indicating a larger difference between the two tasks the higher the spatial frequency. A correlation analysis showed that the correlation between the ratio of the bootstrapped SEs and the spatial frequency was significant and strong (*r* = −0.985, *p* = 0.002). The averaged slope of the best linear fits for individuals’ data (plotted as colored dashed lines in Figure 3) was −0.0684 ± 0.0068 (Mean ± SE), which matched the slope of the best linear fit for the averaged data (plotted as a black solid line), i.e., −0.0685.

**Figure 4:**
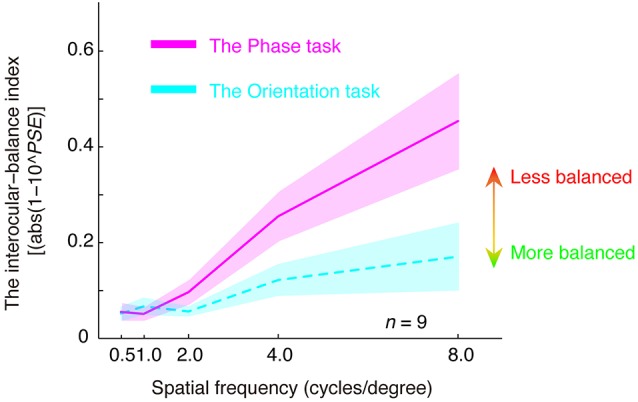
The relationship between the interocular-balance index and spatial frequency. The interocular-balance index was calculated by abs(1–10^PSE^) and plotted as a function of the spatial frequency for the two tasks. Areas indicate the range of ± between subjects’ SEs.

In Figure 4, we plotted the estimated interocular-balance index, calculated by abs(1–10^PSE^), as a function of the spatial frequency for the two tasks. An interocular-balance index of 0 indicates balanced binocular combination, while a larger interocular-balance index indicates a more imbalanced binocular combination (it should be noted that, for the phase task, since observers’ binocular eye balance could not be precisely measured at high spatial frequency, so the averaged interocular-balance index across subjects might also not be precise at high spatial frequency). The results suggest that the two eyes were more imbalanced at high spatial frequencies than they are at low spatial frequencies for both the binocular phase combination and the binocular orientation combination. The spatial frequency dependency was significant for both tasks: for the orientation task, *r* = 0.970, *p* = 0.006; for the phase task, *r* = 0.993, *p* = 0.001; two-tailed Pearson correlation. On the other hand, more imbalance was found in the phase task than that in the orientation task, especially at a high spatial frequency. A repeated-measure analysis of variance (ANOVA) also showed that the interocular-balance index was significantly different between the two tasks (*F*_(1,8)_ = 16.209; *p* = 0.004) and was significantly different between different spatial frequencies (*F*_(1.684,13.47)_ = 9.785; *p* = 0.003; Greenhouse-Geisser method was used to adjust the degrees of freedom), the interaction between the task and the spatial frequency was also significant (*F*_(1.505,12.042)_ = 5.343; *p* = 0.028).

### Experiment 2. Measurement of Binocular Eye Balance With a Larger Interocular Phase Difference

So far, we have shown that the orientation task has superior precision to that of the binocular phase combination paradigm because of its associated smaller SEs of the PSE, especially at high frequencies. Precise results were also achievable at 8 cycles/degree, as observers’ psychometric functions were still sufficiently steep for PSE estimates when using the binocular orientation task, but not when tested using the binocular phase task. During this measurement, a small interocular phase difference at the edge of the gratings of 45° was used to minimize any potential effect of binocular rivalry. The question arises as to whether better results would be obtained using the binocular phase combination task if a larger phase difference was used. Using a 135° interocular phase difference (Ding et al., 2013b), a previous study showed that the psychometric functions are sufficiently steep for PSE measurements to be derived using the binocular phase combination task at 2.72 cycles/degree. We, therefore, conducted an additional control experiment (Experiment 2), in which similar measurements as described in Experiment 1 were undertaken at 8 cycles/degree, while the interocular phase difference at the edge of the gratings increased from 45° to 135°. For the orientation task, this was achieved by applying a larger interocular orientation difference (from 7.2° to 21.7°) while kept the stimulus size fixed. If our observation in Experiment 1 (i.e., smaller SEs of the PSE of the orientation task than that of the phase task) was simply due to the confounding influence of the magnitude of interocular phase difference, we should reveal smaller SEs of the PSE of the phase task than found previously and ones that are comparable to that previously found for the orientation task (Experiment 1).

**Figure 5:**
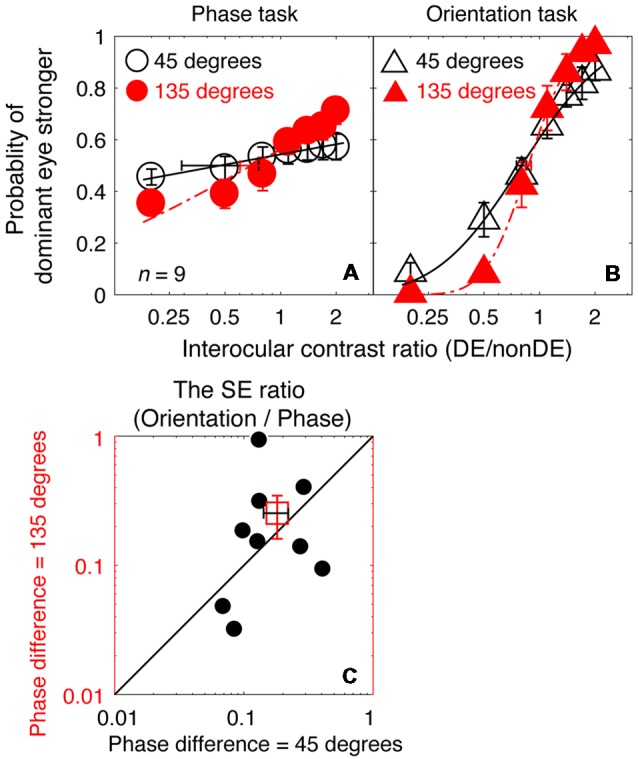
A comparison of the precision of the two tasks at 8 cycles/degree; interocular phase difference of 45° vs. 135°. The average psychometric functions for the two tasks and two interocular phase differences are plotted in **(A,B)**: black circles and black triangles represent the mean probability of dominant eye stronger for the binocular phase combination paradigm and the binocular orientation combination paradigm when the interocular phase difference was 45° (data from Experiment 1); red circles and red triangles represent the mean probability of dominant eye stronger for the binocular phase combination paradigm and the binocular orientation combination paradigm when the interocular phase difference was 135° (data from Experiment 2). The solid and dash line represents the best fitting cumulative distribution functions. Error bars represent SEs across the nine subjects. **(C)** The bootstrapped SE ratio between the orientation task and the phase task at the 135° conditions was plotted as a function of that at the 45° condition for the nine observers. Each open circle represents results of one observer; the filled circle represents the averaged results. The dash line is the equal line. Error bars represent SEs across the nine subjects.

In Figures 5A,B, we plot the averaged psychometric functions for the phase task and the orientation task for the two interocular phase differences. Increasing the interocular phase difference from 45° (black open symbols) to 135° (red filled symbols) resulted in steeper psychometric functions for both tasks. However, the slopes of the orientation task were still higher than those of the phase task for all of our nine observers as evidenced by the smaller bootstrapped SE estimates of the PSE in the orientation task that are shown in Figures 5A,B. We also further compared the SE ratio between the two tasks at the 45° condition with that at the 135° condition (Figure 5C), and found that the difference was not significant (*z* = −0.533, *p* = 0.594, two-tailed Wilcoxon Signed Ranks Test). With these results, there is no support for the proposition that the difference in precision of the PSE estimate that we observed between the two tasks (phase and orientation) in Experiment 1 was due to the small interocular phase difference used.

### Experiment 3. Measurement of Binocular Eye Balance With a Higher Stimuli Resolution

For our measurements in Experiment 1, the gratings were scaled with a fixed two-cycle size at different spatial frequencies, so that the interocular phase difference at the edge of the gratings was fixed. This means that the stimulus resolution (i.e., pixels per cycle) decreased as spatial frequency increased. Even though stimulus resolutions equally affected both the phase task and the orientation task, it will impact the phase measurements more because it will directly limit the resolution of the phase measurement *per se*. To ensure that our conclusions from Experiment 1, namely that the orientation combination task provided a more precise measure of the binocular eye balance than the phase combination task at higher spatial frequencies, we conducted Experiment 3, which was similar to Experiment 1 for the 8 cycles/degree stimulus condition but with a viewing distance increased from 85.5 to 342 cm, so that the stimulus could be displayed at four times its previous spatial resolution.

**Figure 6:**
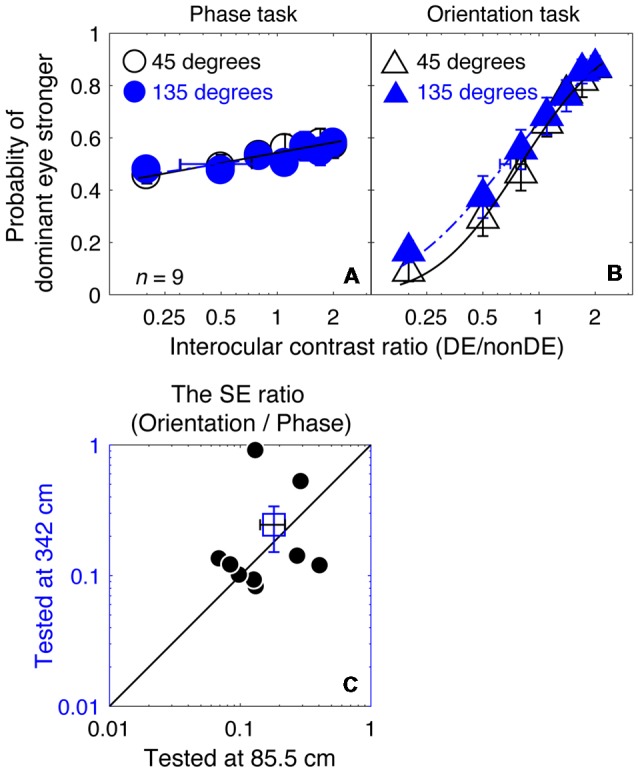
A comparison of the precision of the two tasks at 8 cycles/degree; viewing distance of 85.5 cm vs. 342 cm. The average psychometric functions for the two tasks and two viewing distances are plotted in **(A,B)**: black circles and black triangles represent the mean probability of DE stronger for the binocular phase combination paradigm and the binocular orientation combination paradigm when the viewing distance was 85.5 cm (i.e., a low stimuli resolution; data from Experiment 1); blue circles and red triangles represent the mean probability of DE stronger for the binocular phase combination paradigm and the binocular orientation combination paradigm when the viewing distance was 342 cm (i.e., stimuli resolution increased to four times higher; data from Experiment 3). The solid and dash line represents the best fitting cumulative distribution functions. Error bars represent SEs across the nine subjects. **(C)** The sigma ratio between the orientation task and the phase task at the 342 cm conditions was plotted as a function of that at the 85.5 cm condition for the nine observers. Each open circle represents results of one observer; the filled circle represents the averaged results. The dash line is the equal line. Error bars represent SEs across the nine subjects.

In Figures 6A,B, we plotted the averaged psychometric functions for the phase task and the orientation task at these two viewing distances. However, the slopes of the orientation task were still higher than those of the phase task for all of our nine observers, as evidenced by the smaller bootstrapped SE estimates of the PSE in the orientation task that are shown in Figures 6A,B. This results in bootstrapped SEs for the PSE being much smaller for the orientation than the phase task reflecting the superior precision of the former task. We also compared the bootstrapped SE ratio between the two tasks at the low stimulus resolution condition (@85.5 cm) compared to that at the high stimulus resolution condition (@342 cm) in Figures 6C, and found that the difference was not significant (*z* = −0.178, *p* = 0.859, two-tailed Wilcoxon Signed Ranks Test). With these results, we have no reason to think that the reason why the orientation task provides more precise measurement of the binocular eye balance in Experiment 1 has anything to do with the spatial resolution of the stimuli used.

## Discussion

In this study, we assessed the precision of binocular eye balance measurement using a binocular orientation combination paradigm and compared it with that of the standard binocular phase combination paradigm in normal adults. With our way of “matching” the two tasks (matched interocular phase-shift difference at the left and right edges of the gratings), we show clear evidence that the orientation paradigm is superior to the binocular phase combination paradigm in terms of precision for the measurement of PSE, especially at high frequencies. We also show that normal adults’ two eyes are less balanced at high spatial frequencies than that at low spatial frequencies.

In Experiment 1, 3.6° of counter-clockwise and clockwise oriented (from horizontal) sine-wave gratings were used to measure binocular eye balance for the binocular combination of orientation information. This degree of orientation tilt was chosen so that the phase difference at the left and right ends of the two cycles gratings were 45°, matching that used in the binocular phase combination measurement. Since previous studies have shown that the two eyes can fuse interocular phase differences of up to 135° (Ding and Sperling, 2006; Huang et al., 2009), this choice of orientation tilt ruled out any potential binocular rivalry artifact in the comparison of these two tasks. Furthermore, the 7.2° of interocular orientation difference we used in the current study was well within the effective range of single vision (Braddick, 1979). In Experiment 2, we further tested the binocular phase combination and binocular orientation combination at 8 cycles/degree with three times larger interocular phase-shift difference (i.e., 135°), the largest interocular phase-shift difference that had been validated in previous studies in binocular combination (Ding and Sperling, 2006; Huang et al., 2009). Phase shifts larger than 135°, may result in binocular rivalry.

Given the fact that all monitors have a limited spatial resolution and that this limitation affects higher spatial frequency more, *could such a spatial resolution limitation contribute to the precision difference we found here between these two tasks?* To answer it, we conducted Experiment 3, in which the stimulus resolution was increased by a factor of 4 by changing viewing distance. We show that the difference in precision between the two tasks at higher spatial frequencies remains the same at the longer viewing distance.

The relationship between orientation tuning and spatial frequency has previously been measured using psychophysical tests (Campbell and Kulikowski, 1966; Phillips and Wilson, 1984; Blake and Holopigian, 1985; Baker and Meese, 2007; Cass et al., 2009) and physiology recordings (Maffei et al., 1973; Movshon and Lennie, 1979). Phillips and Wilson (1984), in a masking paradigm, found that the orientation tuning in humans narrowed as the peak spatial frequency of the test stimuli increased. Results from single-unit physiological studies also come to a similar conclusion. In the monkey striate cortex, De Valois et al. (1982) showed that the orientation bandwidth of a cell’s orientation tuning function is likely to be negatively correlated with the cell’s preferred spatial frequency. Additional evidence has been provided by Vidyasagar and Siguenza (1985), who found that with increasing stimulus spatial frequency, the orientation tuning of simple cells becomes progressively narrower in neurons of cat area 17. However, in measuring the spatial frequency and orientation tuning dynamics in awake monkey, Mazer et al. (2002) found that spatial frequency and orientation tuning are largely separable in V1. Nevertheless, these previous studies suggest that there is either an inverse proportionality or a separable dependence of orientation bandwidth on spatial frequency, which may explain why the precision of orientation-based judgments remains high at high spatial frequencies.

Interestingly, we also found that with increasing spatial frequency, observers’ two eyes became more imbalanced. Such a spatial frequency dependency of binocular eye dominance is consistent with and extends Kwon et al.’s (2015) finding in amblyopia using a dichoptic ETDRS letter task acuity chart task from 0.5 to 5.0 cycles/degree and Ding et al.’s (2013a) study in amblyopia using the binocular phase combination from 0.68 to 2.72 cycles/degree. The spatial frequency dependency of binocular eye dominance was true for both the binocular phase combination task and the binocular orientation combination task. However, there was a significant between-task difference, especially at high spatial frequencies. This might be due to the different processing in different binocular combination pathways (Huang et al., 2010, 2011; Zhou et al., 2013).

In clinical practice, anomalies in binocular eye balance are commonly detected by the Worth 4-dot test (Shimko, 2001; Roper-Hall, 2004). There is also quantitative measure using Sbisa bar, Bagolini filter bar, and neutral density filter bar (Piano and Newsham, 2015) to help clinicians in assessing the need for/effect of therapy. In the current study, we show that using a simple orientation discrimination task and constant stimuli measurement method, we are able to precisely quantify the extent of perceptual eye dominance using binocular orientation combination. The main advantage of our measure is that it could provide not only quantitative assessment of the binocular eye balance but also enable precise estimation for stimuli of different spatial frequencies (i.e., up to 8 cycles/degree), which in turn provide more information for understanding the binocular visual deficits in diseases such as amblyopia. This approach may be more time efficient and thus more clinically relevant in assessing binocular eye balance than that of other approaches using signal detection rating methodology (Yehezkel et al., 2016), which are more parametric in nature and ideally suited for answering modeling questions concerning binocular contrast-gain control. In total, approximately 12–15 min were required for each participant to make a precise measurement of one psychometric function for one spatial frequency (constant stimuli method with seven levels of interocular contrast ratio and 80 trials per level). This can be shortened to 4 min for clinical use if only five levels of interocular contrast ratio with 40 trials per level are measured.

We conclude that the binocular orientation combination paradigm provides an efficient and precise way for measuring the binocular eye balance across a wide spatial frequency range and thus could be an effective tool in quantifying binocular function in normal as well as patients with binocular anomalies.

## Data Availability

The data used to support the findings of this study are available from the corresponding author upon request.

## Ethics Statement

This study was carried out in accordance with the recommendations of the ethics committee of the Wenzhou Medical University, with written informed consent from all subjects after explanation of the nature and possible consequences of the study. All subjects gave written informed consent in accordance with the Declaration of Helsinki. The protocol was approved by the ethics committee of the Institutional Review Boards of Wenzhou Medical University.

## Author Contributions

JZ, DS, FL, JQ and RH conceived the experiments. YW, YL, YC, LG, YM and XC performed the experiments. YW, ZH, YL and JZ analyzed the data and interpreted the data. YW, ZH, JZ, and RH wrote the manuscript. All authors contributed to manuscript revision, read and approved the submitted version.

## Conflict of Interest Statement

The authors declare that the research was conducted in the absence of any commercial or financial relationships that could be construed as a potential conflict of interest.

## References

[B1] Baker D. H. Meese T. S. (2007). Binocular contrast interactions: dichoptic masking is not a single process. Vision Res. 47, 3096–3107. 10.1016/j.visres.2007.08.013 17904610

[B2] Bao M. Dong B. Liu L. Engel S. A. Jiang Y. (2018). The best of both worlds: adaptation during natural tasks produces long-lasting plasticity in perceptual ocular dominance. Psychol. Sci. 29, 14–33. 10.1177/0956797617728126 29160741

[B3] Blake R. Holopigian K. (1985). Orientation selectivity in cats and humans assessed by masking. Vision Res. 25, 1459–1467. 10.1016/0042-6989(85)90224-x 4090280

[B4] Braddick O. J. (1979). Binocular single vision and perceptual processing. Proc. R. Soc. Lond. B Biol. Sci. 204, 503–512. 10.1098/rspb.1979.0043 38462

[B5] Brainard D. H. (1997). The psychophysics toolbox. Spat. Vis. 10, 433–436. 10.1163/156856897x00357 9176952

[B6] Campbell F. W. Kulikowski J. J. (1966). Orientational selectivity of the human visual system. J. Physiol. 187, 437–445. 10.1113/jphysiol.1966.sp008101 5972183 PMC1395934

[B7] Cass J. Stuit S. Bex P. Alais D. (2009). Orientation bandwidths are invariant across spatiotemporal frequency after isotropic components are removed. J. Vis. 9, 17.1–17.14. 10.1167/9.12.17 20053108 PMC2927116

[B100] Dane A. Dane S. (2004). Correlations among handedness, eyedness, monocular shifts from binocular focal point, and nonverbal intelligence in university mathematics students. Percept. Mot. Skills. 99, 519–524. 10.2466/pms.99.2.519-524 15560339

[B8] De Valois R. L. Albrecht D. G. Thorell L. G. (1982). Spatial frequency selectivity of cells in macaque visual cortex. Vision Res. 22, 545–559. 10.1016/0042-6989(82)90113-4 7112954

[B10] Ding J. Klein S. A. Levi D. M. (2013a). Binocular combination in abnormal binocular vision. J. Vis. 13:14. 10.1167/13.2.14 23397039 PMC4521338

[B11] Ding J. Klein S. A. Levi D. M. (2013b). Binocular combination of phase and contrast explained by a gain-control and gain-enhancement model. J. Vis. 13:13. 10.1167/13.2.13 23397038 PMC4521337

[B9] Ding J. Sperling G. (2006). A gain-control theory of binocular combination. Proc. Natl. Acad. Sci. U S A 103, 1141–1146. 10.1073/pnas.0509629103 16410354 PMC1347993

[B12] Feng L. Zhou J. Chen L. Hess R. F. (2015). Sensory eye balance in surgically corrected intermittent exotropes with normal stereopsis. Sci. Rep. 5:13075. 10.1038/srep13075 26287935 PMC4541323

[B13] Gambacorta C. Nahum M. Vedamurthy I. Bayliss J. Jordan J. Bavelier D.. (2018). An action video game for the treatment of amblyopia in children: a feasibility study. Vision Res. 148, 1–14. 10.1016/j.visres.2018.04.005 29709618 PMC5984723

[B14] Georgeson M. A. Wallis S. A. (2014). Binocular fusion, suppression and diplopia for blurred edges. Ophthalmic Physiol. Opt. 34, 163–185. 10.1111/opo.12108 24476421 PMC4312971

[B15] Handa T. Mukuno K. Uozato H. Niida T. Shoji N. Shimizu K. (2004). Effects of dominant and nondominant eyes in binocular rivalry. Optom. Vis. Sci. 81, 377–383. 10.1097/01.opx.0000135085.54136.65 15181364

[B16] Handa T. Shimizu K. Uozato H. Shoji N. Ishikawa H. (2012). A new method for quantifying ocular dominance using the balancing technique. Am. Orthopt. J. 62, 77–86. 10.3368/aoj.62.1.77 22848115

[B17] Handa T. Uozato H. Higa R. Nitta M. Kawamorita T. Ishikawa H.. (2006). Quantitative measurement of ocular dominance using binocular rivalry induced by retinometers. J. Cataract Refract. Surg. 32, 831–836. 10.1016/j.jcrs.2006.01.082 16765802

[B19] Hess R. F. Hutchinson C. V. Ledgeway T. Mansouri B. (2007). Binocular influences on global motion processing in the human visual system. Vision Res. 47, 1682–1692. 10.1016/j.visres.2007.02.005 17442362

[B18] Hess R. F. Thompson B. (2015). Amblyopia and the binocular approach to its therapy. Vision Res. 114, 4–16. 10.1016/j.visres.2015.02.009 25906685

[B21] Huang C. B. Zhou J. Lu Z. L. Feng L. Zhou Y. (2009). Binocular combination in anisometropic amblyopia. J. Vis. 9, 17.1–17.16. 10.1167/9.3.17 19757956 PMC2861488

[B20] Huang C. B. Zhou J. Lu Z. L. Zhou Y. (2011). Deficient binocular combination reveals mechanisms of anisometropic amblyopia: signal attenuation and interocular inhibition. J. Vis. 11:4. 10.1167/11.6.4 21546609 PMC3324098

[B22] Huang C. B. Zhou J. Zhou Y. Lu Z. L. (2010). Contrast and phase combination in binocular vision. PLoS One 5:e15075. 10.1371/journal.pone.0015075 21151558 PMC3000330

[B23] Kwon M. Lu Z. L. Miller A. Kazlas M. Hunter D. G. Bex P. J. (2014). Assessing binocular interaction in amblyopia and its clinical feasibility. PLoS One 9:e100156. 10.1371/journal.pone.0100156 24959842 PMC4069064

[B24] Kwon M. Wiecek E. Dakin S. C. Bex P. J. (2015). Spatial-frequency dependent binocular imbalance in amblyopia. Sci. Rep. 5:17181. 10.1038/srep17181 26603125 PMC4658600

[B25] Li J. Thompson B. Deng D. Chan L. Y. Yu M. Hess R. F. (2013). Dichoptic training enables the adult amblyopic brain to learn. Curr. Biol. 23, R308–R309. 10.1016/j.cub.2013.01.059 23618662

[B26] Maffei L. Fiorentini A. Bisti S. (1973). Neural correlate of perceptual adaptation to gratings. Science 182, 1036–1038. 10.1126/science.182.4116.1036 4748674

[B27] Mazer J. A. Vinje W. E. McDermott J. Schiller P. H. Gallant J. L. (2002). Spatial frequency and orientation tuning dynamics in area V1. Proc. Natl. Acad. Sci. U S A 99, 1645–1650. 10.1073/pnas.022638499 11818532 PMC122244

[B28] Movshon J. A. Lennie P. (1979). Pattern-selective adaptation in visual cortical neurones. Nature 278, 850–852. 10.1038/278850a0 440411

[B29] Ooi T. L. He Z. J. (2001). Sensory eye dominance. Optometry 72, 168–178. 11294588

[B30] Ooi T. L. Su Y. R. Natale D. M. He Z. J. (2013). A push-pull treatment for strengthening the ‘lazy eye’ in amblyopia. Curr. Biol. 23, R309–R310. 10.1016/j.cub.2013.03.004 23618663 PMC6485254

[B31] Pelli D. G. (1997). The VideoToolbox software for visual psychophysics: transforming numbers into movies. Spat. Vis. 10, 437–442. 10.1163/156856897x00366 9176953

[B32] Phillips G. C. Wilson H. R. (1984). Orientation bandwidths of spatial mechanisms measured by masking. J. Opt. Soc. Am. A 1, 226–232. 10.1364/josaa.1.000226 6423788

[B33] Piano M. Newsham D. (2015). A pilot study examining density of suppression measurement in strabismus. Strabismus 23, 14–21. 10.3109/09273972.2014.1002621 25790154

[B34] Roper-Hall G. (2004). The “worth” of the worth four dot test. Am. Orthopt. J. 54, 112–119. 10.3368/aoj.54.1.112 21149094

[B35] Shimko J. F. (2001). Binocular vision and ocular motility theory and management of strabismus Gunter K. vonNoorden, M.D.; Emilio C. Campos, M.D. Mosby Inc., Sixth Edition 2002, $149.00; 631 pages, 315 illustrations. Am. Orthopt. J. 51, 161–162. 10.3368/aoj.51.1.161

[B36] Spiegel D. P. Baldwin A. S. Hess R. F. (2016). The relationship between fusion, suppression, and diplopia in normal and amblyopic vision. Invest. Ophthalmol. Vis. Sci. 57, 5810–5817. 10.1167/iovs.16-20438 27802486

[B37] Vidyasagar T. R. Siguenza J. A. (1985). Relationship between orientation tuning and spatial frequency in neurones of cat area 17. Exp. Brain Res. 57, 628–631. 10.1007/bf00237851 3979503

[B38] Yehezkel O. Ding J. Sterkin A. Polat U. Levi D. M. (2016). Binocular combination of stimulus orientation. R. Soc. Open Sci. 3:160534. 10.1098/rsos.160534 28018641 PMC5180139

[B39] Zhou J. Feng L. Lin H. Hess R. F. (2016). On the maintenance of normal ocular dominance and a possible mechanism underlying refractive adaptation. Invest. Ophthalmol. Vis. Sci. 57, 5181–5185. 10.1167/iovs.16-19696 27699413

[B40] Zhou J. Georgeson M. A. Hess R. F. (2014a). Linear binocular combination of responses to contrast modulation: contrast-weighted summation in first- and second-order vision. J. Vis. 14:24. 10.1167/14.13.24 25424859

[B42] Zhou J. Liu R. Zhou Y. Hess R. F. (2014b). Binocular combination of second-order stimuli. PLoS One 9:e84632. 10.1371/journal.pone.0084632 24404180 PMC3880315

[B41] Zhou J. Huang P.-C. Hess R. F. (2013). Interocular suppression in amblyopia for global orientation processing. J. Vis. 13:19. 10.1167/13.5.19 23608341

