# Corrigendum: The Binocular Balance at High Spatial Frequencies as Revealed by the Binocular Orientation Combination Task

**DOI:** 10.3389/fnhum.2019.00196

**Published:** 2019-06-12

**Authors:** Yonghua Wang, Zhifen He, Yunjie Liang, Yiya Chen, Ling Gong, Yu Mao, Xiaoxin Chen, Zhimo Yao, Daniel P. Spiegel, Jia Qu, Fan Lu, Jiawei Zhou, Robert F. Hess

**Affiliations:** ^1^The First Affiliated Hospital of Wenzhou Medical University, Wenzhou, China; ^2^School of Ophthalmology and Optometry and Eye Hospital, Wenzhou Medical University, Wenzhou, China; ^3^State Key Laboratory of Ophthalmology, Optometry and Vision Science, Wenzhou Medical University, Wenzhou, China; ^4^Vision Sciences, Essilor R&D, Center for Innovation and Technology, Singapore, Singapore; ^5^McGill Vision Research, Department of Ophthalmology, McGill University, Montreal, QC, Canada

**Keywords:** binocular eye dominance, spatial frequency, binocular orientation combination, binocular phase combination, contrast-gain

An author name was incorrectly spelled as “Zhifeng He.” The correct spelling is “Zhifen He.”

The authors apologize for this error and state that this does not change the scientific conclusions of the article in any way. The original article has been updated.

